# A Case Report: Pseudoangiomatous Stromal Hyperplasia Tumor Presenting as a Palpable Mass

**DOI:** 10.3389/fsurg.2015.00073

**Published:** 2016-01-19

**Authors:** Q. D. Vo, G. Koch, J. M. Girard, L. Zamora, Jean Bouquet de Jolinière, F. Khomsi, A. Feki, H. M. Hoogewoud

**Affiliations:** ^1^Department of Radiology, HFR Fribourg, Cantonal Hospital, Fribourg, Switzerland; ^2^Department of Gynecology and Obstetrics, HFR Fribourg, Cantonal Hospital, Fribourg, Switzerland

**Keywords:** pseudoangiomatous stromal hyperplasia, breast tumor, MRI, phyllode tumor, mammography

## Abstract

We report a case of woman with a palpable lump on her left breast. On mammography, a huge mass located between the inner and the outer inferior breast quadrants of the left breast was found. The ultrasound examination realized later revealed a heterogeneous mass with smooth and lobulated borders. An MRI was also performed, showing an oval mass with heterogeneous areas of enhancement. Finally, a core biopsy under sonographic guidance revealed a pseudoangiomatous stromal hyperplasia of the breast.

## Background

Pseudoangiomatous stromal hyperplasia (PASH) is a relatively common finding on histological examination and can be found in normal breasts. It presents rarely as a palpable lump. The main differential diagnosis on imaging is fibroadenoma and phyllode tumor. Histologically, it is important to differentiate PASH from a low-grade angiosarcoma. Management of PASH differs according to presentation and clinical symptoms. The main purpose of this article is to present imaging features and the management of PASH.

## Case Presentation

A 50-year-old woman reported a palpable mass in her left breast for few months associated with radiating pain in her left arm. Her medical history revealed no previous pregnancy, no previous pathology in the family, a tubal ligation with Pfannenstiel technique in Brazil in 1986, and a total hysterectomy with conservation of ovaries due to fibroids in 2011. The physical examination confirmed a palpable hard mass in the left breast located in the inferior quadrants. No palpable lymph nodes were noticed.

## Investigations

**Figure 1:**
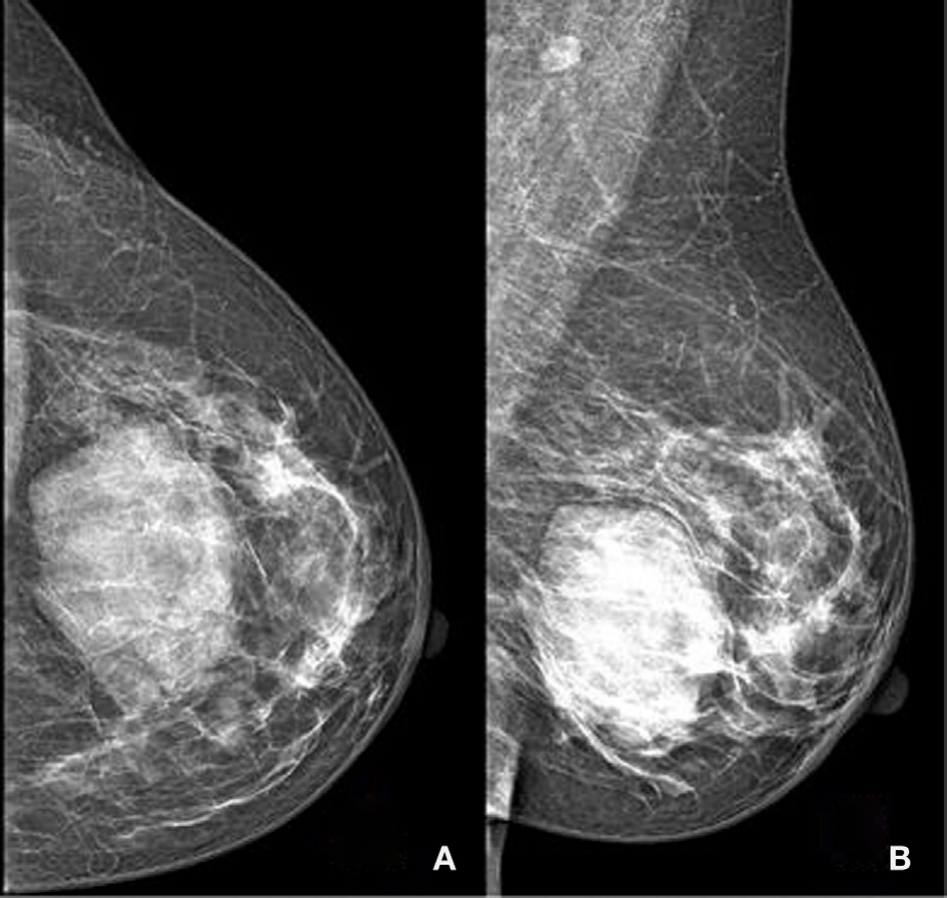
**Mammography performed with craniocaudal (A) and mediolateral oblique (B) incidences of the left breast shows a huge well-defined mass located between the inner and the outer inferior quadrants, with no suspicious features**.

**Figure 2:**
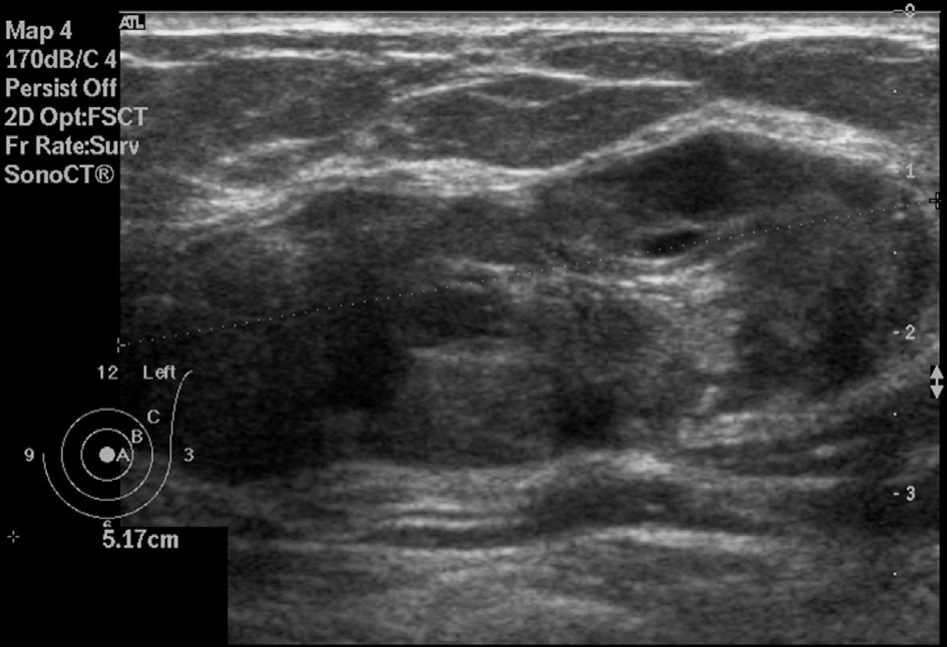
**Ultrasound of the left breast centered on the palpable lump showing an oval, heterogeneous lesions with smooth borders**.

**Figure 3:**
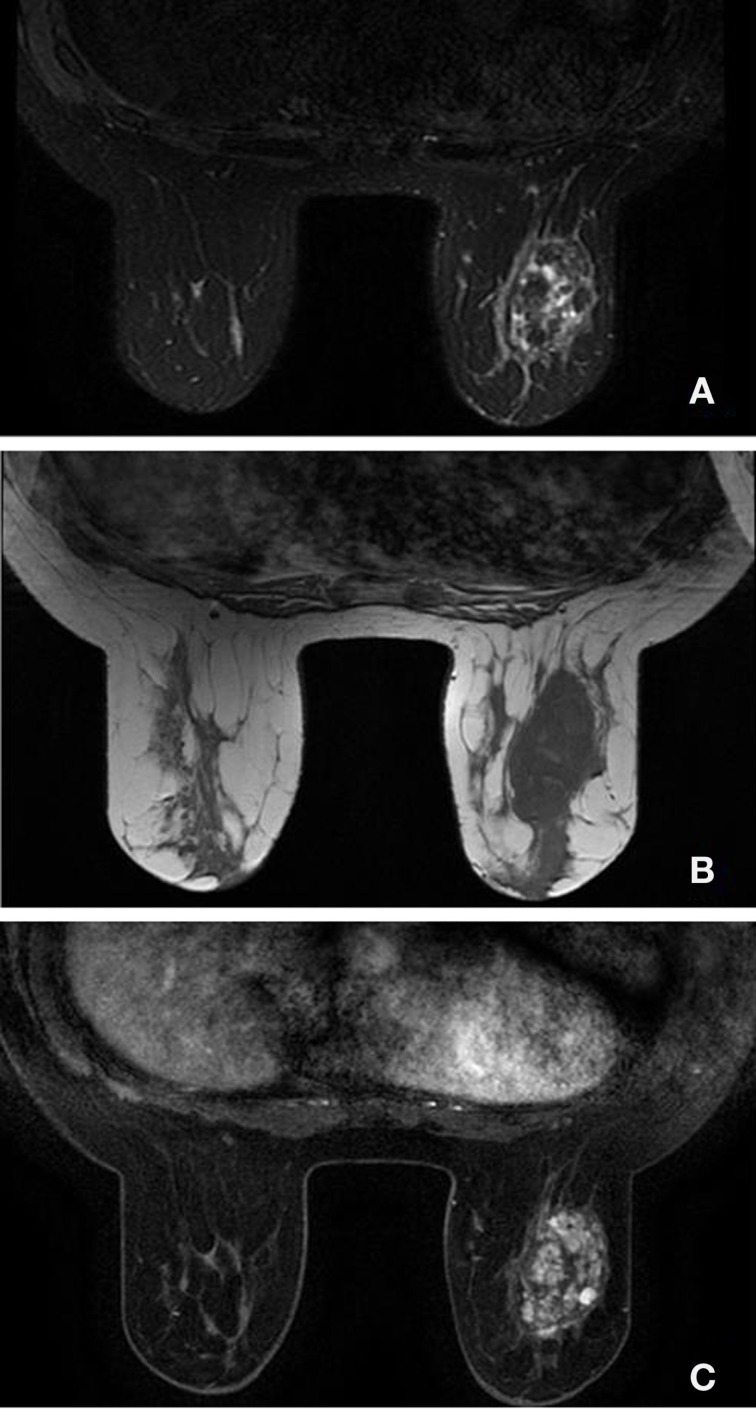
**MRI showing the mass on the left breast with lobulated appearances**. On T2-weighted MR image with fat saturation **(A)**, the lesion demonstrates a heterogeneous appearance with areas of low and high signal. On T1-weighted MR image **(B)**, the lesion shows a more homogeneous appearance with a hyposignal. On T1-weighted MR image with gadolinium and fat saturation **(C)**, the mass demonstrates a heterogeneous enhancement, with a polylobulated appearance. No lymphadenopathies are seen.

On mammography (Figure 1), including a craniocaudal and a mediolateral oblique view of the breasts, a huge well-demarcated mass with lobulated borders containing tiny calcifications in the left breast, corresponding to BI-RADS category 3 was found. This mass was located between the inner and the outer inferior quadrants. An additional ultrasound (Figure 2) was realized and demonstrated a well-defined heterogeneous lobulated hypoechoic mass measuring 5.17 cm × 1.74 cm. In order to confirm the benign nature of the lesion, an MRI scan (Figure 3) was performed, which demonstrated a huge mass with cystic components presenting a hypointense signal on T1- and T2-weighted images and a heterogeneous enhancement on post-contrast sequences.

## Differential Diagnosis

According to the clinical results of examination and imaging, the main differential diagnosis was a fibroadenoma and phyllode tumor.

## Management

**Figure 4:**
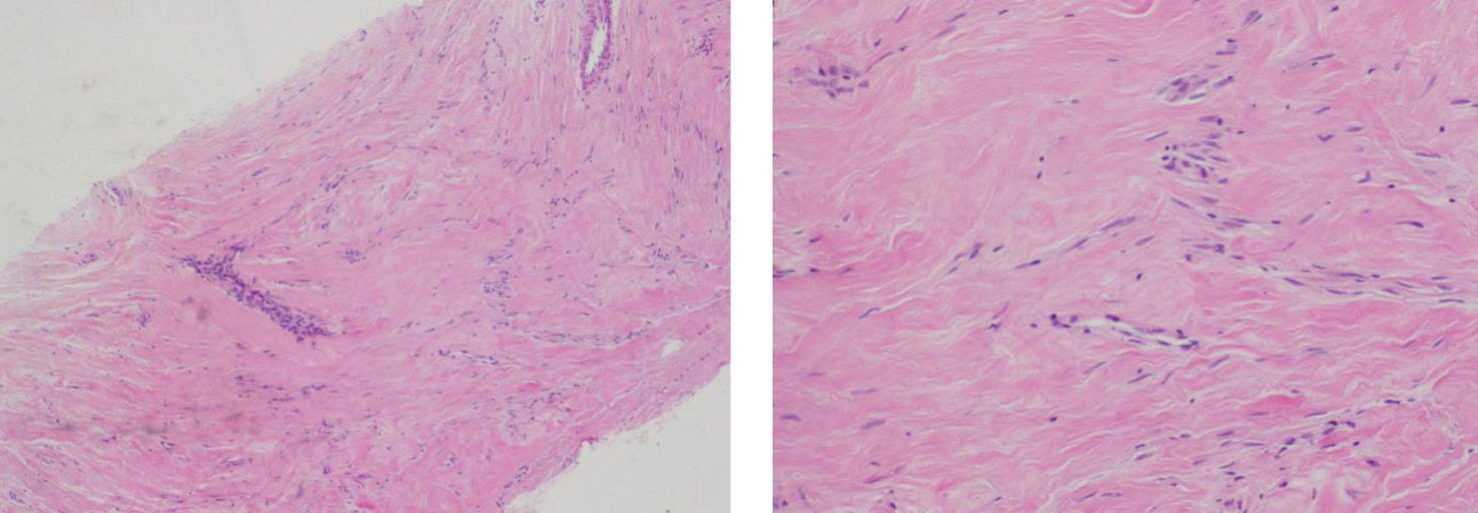
**Microscopic examination showing a pseudo vascular proliferation delineated with endothelial cells with canalar hyperplasia without atypia, without invasion of parenchyma**.

A core biopsy under ultrasound guidance was performed. The specimen revealed a pseudo vascular proliferation of mammary stroma delineated by endothelial cells with canalar hyperplasia without atypia (Figure 4). These findings were compatible with a PASH. After discussion with the patient, it was decided to follow-up the lesion.

## Discussion

Pseudoangiomatous stromal hyperplasia is a benign proliferative breast disease that was first described by Vuitch et al. (1). This lesion is characterized by a dense, collagenous proliferation of mammary stroma, forming inter-anastomosing capillary-like spaces. It is thought that hormonal factors play an important role in PASH (2). According to Anderson et al., this lesion represents an important hyper-response to progesterone and estrogen (3). PASH is a common histological finding in breast biopsy specimens and can also be found in a normal breast that is in association with proliferative or non-proliferative fibrocystic changes (4), but it is rarely a symptomatic lesion. Clinically, PASH can presents as a solitary firm, mobile, palpable lump, or as multifocal nodules in 60% of cases (1, 2, 4–6) or can be discovered incidentally on imaging. PASH can be found in teenage girls as well as in postmenopausal women with or without hormonal therapy replacement (6, 7). It is important to recognize this entity because it can be easily confused with others benign tumors, such as fibroadenoma, phyllode tumor, or with malignant tumors, such as angiosarcoma (1, 6). Unfortunately, imaging features of PASH are non-specific (4). On mammography, the most common appearance described is a well-defined, uncalcified mass, with regular borders. Spiculated borders, suspicious borders, and architectural distortion can also be seen but are uncommon (8–10). On ultrasound, PASH tends to be an oval, round hypoechoic mass or can presents as a heterogeneous mass with cystic areas (8). According to Cohen et al. (4), when a focal lesion with well-defined borders, containing no calcifications on mammography or a well-defined hypoechoic mass on ultrasound is seen, PASH can be considered and included in the differential diagnosis.

Clinically and on imaging, the differential diagnosis include fibroadenoma, especially in young patient and a phyllode tumor in older women (1, 5, 7). Histologically, PASH can be very similar to low-grade angiosarcoma.

Definitive diagnosis is based on histology (11–13). As mentioned earlier, PASH can have very similar histological features as low-grade angiosarcoma. But unlike low-grade angiosarcoma, PASH lacks of invasive features (14) and contains no necrosis, mitoses, and no destruction of mammary epithelial structures (1).

Management of PASH depends on presentation. When PASH is incidentally discovered or when it is asymptomatic, it can be followed up yearly by ultrasound or mammography for a period of 36 months (11, 12, 15). Surgical procedures are indicated for symptomatic lesion with mechanical complaints, pain or apprehension for an alternative malignant lesion (11, 12).

## Ethics Statement

Written informed consent was obtained from the patient prior to presenting the case.

## Author Contributions

Each of the authors QV, GK, JG, LZ, JB, FK, AF and HH, participated in caring for the patient. QV is the principal author.

## Conflict of Interest Statement

The authors declare that the research was conducted in the absence of any commercial or financial relationships that could be construed as a potential conflict of interest. The authors confirm no ethical conflict with the patient. The handling Editor Issam Lebbi declares that, despite having collaborated with the authors, A. Feki and Jean Bouquet de Jolinière, the review process was handled objectively.
